# Physical and mental health comorbidity is common in people with multiple sclerosis: nationally representative cross-sectional population database analysis

**DOI:** 10.1186/1471-2377-14-128

**Published:** 2014-06-13

**Authors:** Robert J Simpson, Gary McLean, Bruce Guthrie, Frances Mair, Stewart W Mercer

**Affiliations:** 1 General Practice and Primary Care, Institute of Health and Wellbeing, University of Glasgow, Glasgow G12 9LX, Scotland, UK; 2 Quality, Safety and Informatics Research Group, Population Health Sciences Division, Mackenzie Building, Kirsty Semple Way, Dundee DD2 4BF, Scotland, UK

## Abstract

**Background:**

Comorbidity in Multiple Sclerosis (MS) is associated with worse health and higher mortality. This study aims to describe clinician recorded comorbidities in people with MS.

**Methods:**

39 comorbidities in 3826 people with MS aged ≥25 years were compared against 1,268,859 controls. Results were analysed by age, gender, and socioeconomic status, with unadjusted and adjusted Odds Ratios (ORs) calculated using logistic regression.

**Results:**

People with MS were more likely to have one (OR 2.44; 95% CI 2.26-2.64), two (OR 1.49; 95% CI 1.38-1.62), three (OR 1.86; 95% CI 1.69-2.04), four or more (OR 1.61; 95% CI 1.47-1.77) non-MS chronic conditions than controls, and greater mental health comorbidity (OR 2.94; 95% CI 2.75-3.14), which increased as the number of physical comorbidities rose. Cardiovascular conditions, including atrial fibrillation (OR 0.49; 95% CI 0.36-0.67), chronic kidney disease (OR 0.51; 95% CI 0.40-0.65), heart failure (OR 0.62; 95% CI 0.45-0.85), coronary heart disease (OR 0.64; 95% CI 0.52-0.71), and hypertension (OR 0.65; 95% CI 0.59-0.72) were significantly less common in people with MS.

**Conclusion:**

People with MS have excess multiple chronic conditions, with associated increased mental health comorbidity. The low recorded cardiovascular comorbidity warrants further investigation.

## Background

Multiple Sclerosis (MS) is a chronic, debilitating disease that can cause widespread damage to the nervous system, with potentially devastating physical, psychological, vocational, and social consequences [1] and because of its progressively disabling nature significant health care and social costs accumulate [2]. Scotland has the highest prevalence of MS worldwide, with a number of potential aetiological factors implicated, including genetics, infection, low sun exposure, and diet [3-5].

Comorbidity is common in MS, and particular patterns of comorbid disease predominate. Given the high level of medical contact and input that people with MS can require, surveillance bias may be a factor in these findings, although clear-cut research data appears lacking in this regard. Comorbidity can be defined as any additional condition (over and above MS) requiring treatment, or which alters organ function [6]. Common comorbid conditions include anxiety, depression, and bipolar disorder, as well as a variety of physical conditions, including autoimmune disorders, gastrointestinal dysfunction, arthritis, visual impairment, and possibly cardiovascular (CV) diseases [7-12]. However, studies have ranged in terms of sampling, sample size, and the number and type of comorbidities recorded.

In Taiwan, using a modified version of the Elixhauser Comorbidity Index, Kang et al. [11] reported on findings from a national health insurance research database on 30 comorbidities amongst a group of 898 patients with MS compared against 4490 randomly matched controls. They found that people with MS had higher rates of both psychiatric (depression, dementia, psychoses) and various medical (neurological, pulmonary, endocrine, autoimmune, renal, gastrointestinal, infectious, haematological, and oncological) comorbidities. In Canada, Warren et al. [10] used the Canadian Community Health Survey to extract comorbidity data for people with MS, and examined the prevalence of 21 self-reported comorbdities in 335 individuals including medical and psychiatric complaints. They found that the mean number of comorbidities reported was 1.6, and that 10% of respondents reported 8 or more [10]. In the United States (US), Marrie et al. [9] assessed the prevalence of 33 self-reported comorbidities in 8983 people with MS aged 16–60 which comprised 7-10% of the US MS population (although was not nationally representative). They reported that multiple comorbidity was present in 77.1% (6907/8983) of participants, with 30.4% (n = 2907) having one comorbidity, 25.6% (n = 1766) having two, and 44.1% (n = 3044) having three or more. Marrie et al. [9] found that comorbidity was more likely amongst men, the older aged, those of lower socio-economic status, and those of African American ethnicity. The comorbidities included in the Marrie et al. [9] study were reported as being selected on the basis of their being either frequently reported in the general population, or frequently reported amongst those with MS. The respondents to the survey were mostly female (75.8%) and Caucasian (94.3%), with the study authors noting that non-responders were significantly more likely to be younger, of lower socio-economic status, non-Caucasian ethnicity, and more disabled.

The aim of this study is to describe a wide range of clinician recorded physical and mental comorbidities (n = 39) in people with MS, compared to controls, in a large and nationally representative primary care population in Scotland.

## Methods

Analysis used cross-sectional data from a nationally representative Scottish Primary Care dataset, supplied by the Primary Care Clinical Informatics Unit, University of Aberdeen, UK, as part of a programme of research on multimorbidity [13]. The anonymous use of this data has prior approval from the United Kingdom National Health Service National Research Ethics Service [13]. The dataset holds clinical information on 1,751,841 individuals, registered with 314 general practices across Scotland, which was extracted in April 2007, and is a complete copy of all historical data at that point. The patient data is for approximately one third of the Scottish population and is nationally representative in terms of age, gender, and socioeconomic status. In Scotland, all general practices use electronic records, and have done so extensively since the late 1990’s for managing their registered lists, for prescribing, and for the recording of morbidities using a standard coding system (Read Codes). MS was defined as the presence ever of a Read Code for MS using a code-set created by NHS Scotland Information Services Division [14]. Thirty-nine other chronic conditions were defined, as reported previously [13], including 8 mental health conditions and 31 other physical health conditions. The 40 conditions included (39 others plus MS) were based on the recording by a clinician of a relevant Read Code in an individual’s electronic clinical record and/or on prescription of relevant drugs. The conditions were derived from a combination of recent systematic review evidence (11 conditions), diseases included in the UK General Practice contract Quality and Outcomes Framework (16 conditions), and those specified by NHS Scotland as important for health service planning (26 conditions) (see [13] for details). Extracted data included information on: age, gender, and socioeconomic status (Carstair’s score based on post codes) [15]. Due to the low numbers of people with MS under the age of 25 years, the analysis was restricted to all people aged ≥25 years. Analysis is therefore based on data for 1,268,859 individuals.

### Statistical analysis

Analysis was descriptive and compared comorbidity in people with and without MS. T tests were used to examine differences in mean number of morbidities, and chi-squared tests to examine differences in the percentage of people with comorbidity across variables. Binary logistic regression examined associations between comorbidities in people with MS versus controls. Odds ratios (ORs) adjusted for age, sex and deprivation, with 95% confidence intervals, are reported for people with MS versus the general population. All analysis was undertaken using SPSS v19.

## Results

Of the 1,268,859 individuals in the analysis, 3826 (0.3%; 95% CI 0.29-0.31) had a diagnosis of MS, of which 2767 (72.3%) were female. People with MS were on average slightly older than the unaffected population (mean age 53.4 years versus 51.2 years for controls; p < 0.001). People with MS were also marginally less socioeconomically deprived, as measured by the Carstair’s Score (mean value -0.64 versus -0.22; p < 0.001) (see Table 1). After adjusting for age, sex, and deprivation, those with MS were significantly more likely to have greater than one other chronic condition (OR 2.44; 95% CI 2.26-2.64) (see Table 2).

**Table 1:** General characteristics: MS vs non-MS

	**MS (>25yrs)**	**Non-MS (>25yrs)**	**Significance p**
	**n (%)**	**n (%)**	
**Total**	3,826	1,268,859	
**Gender (% female)**	2,767 (72.3)	647,836 (51.1)	*p<0.0001*
**Mean age (SD)**	53.4 (12.8)	51.2 (16.6)	*p<0.0001*
**Mean deprivation (SD)**	-0.64 (3.0)	-0.2 (3.3)	*p<0.0001*
**Age group**			
**25-44**	1018 (26.6)	507371 (40.0)	*p<0.0001*
**45-64**	2083 (54.4)	471044 (37.1)	*p<0.0001*
**65-84**	685 (17.9)	253915 (20.0)	*p=0.0006*
**85+**	40 (1.0)	36529 (2.9)	*p<0.0001*
**Carstair’s deprivation Quintile (min-max score)**			
**Q1 least deprived**	813 (21.2)	243545 (19.2)	*p=0.0006*
**Q2**	889 (23.2)	274878 (21.7)	*p=0.009*
**Q3**	913 (23.9)	289037 (22.8)	p=0.06
**Q4**	687 (18.0)	241207 (19.0)	*p=0.049*
**Q5 most deprived**	524 (13.7)	220192 (17.4)	*p<0.0001*

**Table 2:** Comorbidity prevalence and type in people with and without MS

	**MS n (%)**	**Non-MS n (%)**	OR* (95% CI)
			**Significance p**
**No comorbidity**	1,027 (26.8)	597,363 (47.1)	0.20 (0.18-0.22) *p<0.001*
**One**	2,799 (73.2)	671,496 (52.9)	2.44 (2.26-2.64) *p<0.0001*
**Two**	714 (18.7)	159,293 (12.6)	1.49 (1.38-1.62) *p<0.0001*
**Three**	546 (14.3)	97,368 (7.7)	1.86 (1.69-2.04) *p<0.0001*
**Four or more**	669 (17.5)	136,847 (10.8)	1.61 (1.47-1.77) *p<0.0001*
**Any physical comorbidity**	2,475 (64.7)	600,669 (47.3)	2.05 (1.90-2.21) *p<0.0001*
**Any mental health comorbidity**	1,541 (40.3)	227,361 (17.9)	2.94 (2.75-3.14) *p<0.0001*

*Odds Ratios adjusted for Age, Sex and Deprivation Scores.

There was no difference found between women and men with MS, with respect to: age, deprivation, or total physical comorbidity count (results not shown). Female patients were significantly more likely to have a mental comorbidity than males (OR 1.26; 95% CI 1.09-1.46), after adjusting for age and deprivation (see Table 3).

**Table 3:** Gender differences in co-morbidity in people with MS

**Physical conditions (Including MS)**	**MS Population characteristics**		
	**Female**	**Male**	OR* (95% CI)
	**n (%)**	**n (%)**	**Significance p**
**2+**	1801(64.6)	678 (63.7)	1.07 (0.91 1.25) p=0.37
**3+**	1021 (36.6)	401 (37.7)	0.98 (0.84 1.14) p=0.55
**Mental conditions**			
**At least 1**	1156 (41.8)	385 (36.4)	1.26 (1.09 1.46) *p=0.02*
**2+**	112 (4.0)	55 (5.2)	1.02 (0.91 1.13) p=0.69

*Odds Ratios adjusted for Age and Deprivation Scores.

**Figure 1:**
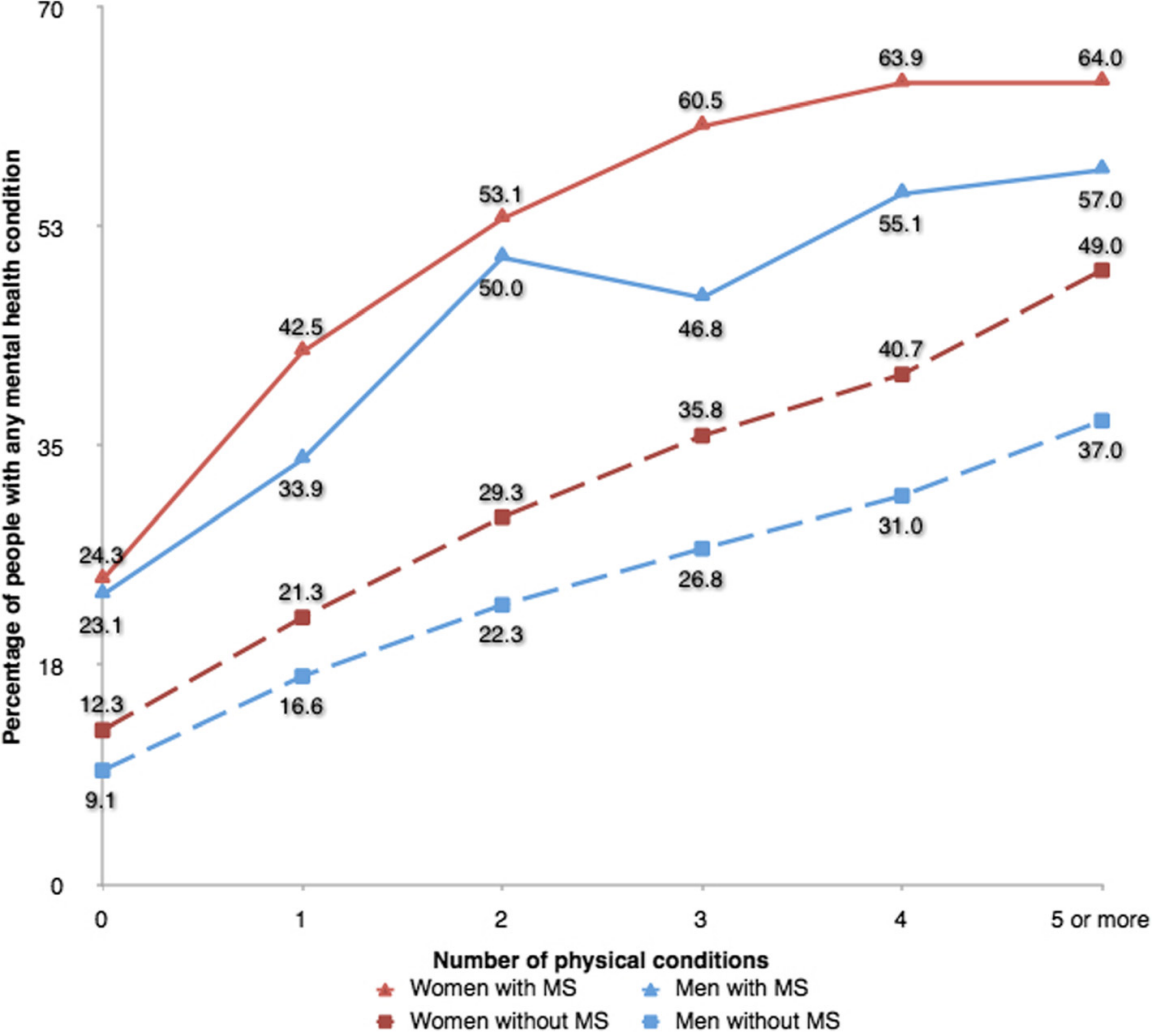
Association between number of physical conditions and presence of any mental health condition.

People with MS had higher levels of chronic conditions after controlling for age, sex and deprivation (OR 2.44; 95% CI 2.26-2.64) and especially mental health comorbidity (OR 2.94; 95% CI 2.75-3.14). People with MS were more likely to have one (OR 2.44; 95% CI 2.26-2.64), two (OR 1.49; 95% CI 1.38-1.62), three (OR 1.86; 95% CI 1.69-2.04), or four or more (OR 1.61; 95% CI 1.47-1.77) non-MS chronic conditions than controls (see Table 2), and as the number of physical morbidities rose, both men and women with MS were consistently more likely to have a mental health comorbidity than the controls (see Figure 1).

### Comorbidity of mental conditions in people with MS

**Figure 2:**
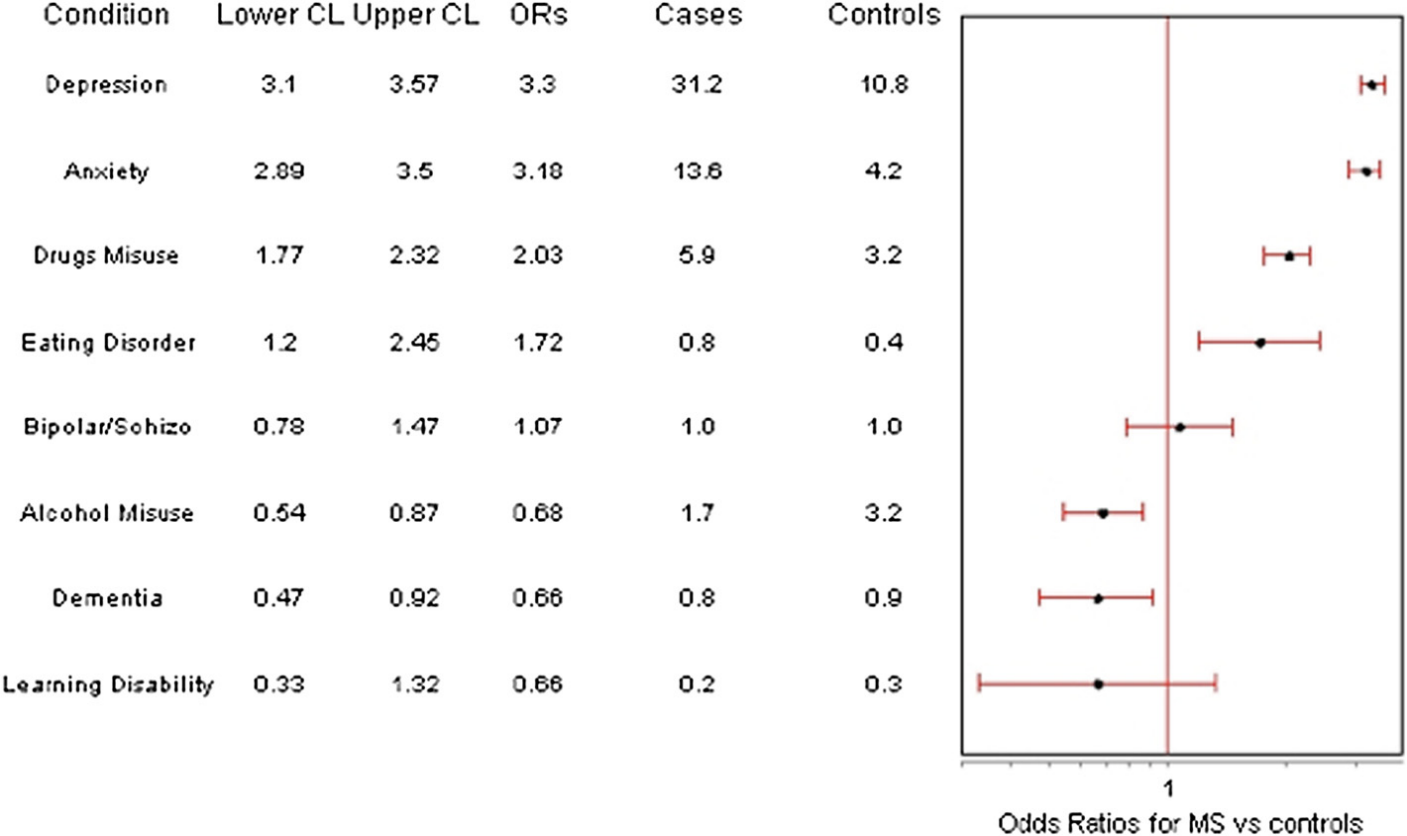
Odds ratios for individual mental conditions: people with MS (n = 3826) vs controls (n = 1,268,859). *Odds Ratios adjusted for Age, Sex and Deprivation Scores. Note on abbreviations [13]: Depression = Read code recorded in last 12 months OR ≥4 anti-depressant prescriptions (excluding low dose tricyclics) in last 12 months; Anxiety = Anxiety & other neurotic, stress related & somatoform disorders, Read code in last 12 months OR ≥ 4 anxiolytic/hypnotic prescriptions in last 12 months. OR ≥4 10/25 mg amitriptyline in last 12 months & do not meet the criteria for ‘Pain.’; Drugs Misuse = Other psychoactive substance misuse; Eating Disorder = Anorexia or Bulimia; Bipolar/Schizo = Schizophrenia (and related non-organic psychosis) or bipolar disorder, Read code ever recorded/recorded in last 12 months (code dependent) OR Lithium prescribed in last 168 days.

People with MS had a higher prevalence for four of the eight mental conditions examined after adjustment for age, sex and socioeconomic status (Figure 2); being highest for depression (OR 3.33; 95% CI 3.10-3.57), followed by anxiety (OR 3.18; 95% CI 2.89-3.50), and problematic drug use (OR 2.03; 95% CI 1.77-2.32) (a mixed category that includes a range of problems associated with prescription and non-prescription drug use). A diagnosis of an eating disorder was more prevalent (OR 1.72; 95% CI 1.20-2.45), but the numbers within the eating disorder group were very small (n = 31). There was no statistically significant difference in prevalence of schizophrenia/bipolar disorder (OR 1.07; 95% CI 0.78-1.47) or learning disability (OR 0.66; 95% CI 0.33-1.32), whilst dementia (OR 0.66; 95% CI 0.47-0.94), and alcohol misuse (OR 0.68; 95% CI 0.54-0.87) were significantly less common amongst people with MS.

### Comorbidity of physical conditions in people with MS

**Figure 3:**
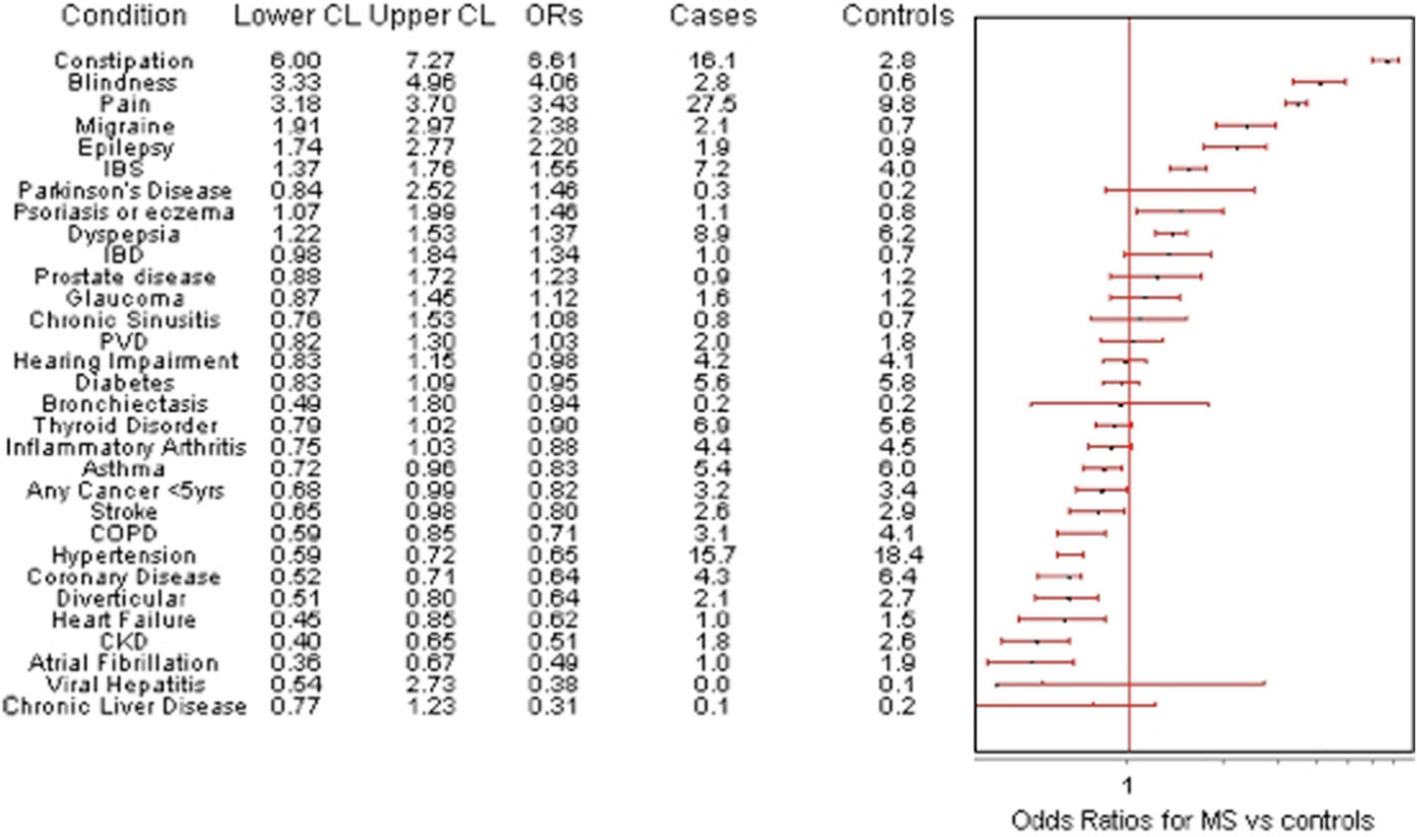
Odds ratios for individual physical conditions: people with MS (n = 3826) vs controls (n = 1,268,859). *Odds Ratios adjusted for Age, Sex and Deprivation Scores. Note on abbreviations [13]: Constipation = ≥4 laxative prescriptions in last year; Pain = ≥4 prescription only medicine analgesic prescriptions in last 12 months OR ≥4 specified anti-epileptics in the absence of an epilepsy Read code in last 12 months; Migraine = ≥4 prescription only medicine anti-migraine prescriptions in last year; Epilepsy = Read code ever recorded AND antiepileptic prescription in last 12 months; IBS = Read code ever recorded OR ≥ 4 prescription only medicine antispasmodic prescription in last 12 months; Parkinson’s Disease = Parkinson’s Disease and Parkinsonism; Psoriasis or Eczema = Read code ever recorded AND ≥ 4 related prescriptions in last 12 months (excluding simple emollients) Dyspepsia = ≥ 4 prescriptions in last 12 months excluding antacids AND NOT (≥4 NSAIDS OR ≥4 aspirin/clopidogrel) IBD = inflammatory bowel disease; PVD = Peripheral vascular disease; Stroke = Stroke and Transient ischaemic attack; COPD = Chronic obstructive pulmonary disease; CKD = Chronic kidney disease; Inflammatory Arthritis = Rheumatoid arthritis, other inflammatory polyarthropathies & systematic connective tissue disorders; Asthma = Read code ever recorded AND any prescription in last 12 months.

Figure 3 shows that of the thirty physical conditions assessed, the prevalence was higher in the MS group than controls for ten conditions, lower for eleven, and no different for nine conditions. Prevalence was highest in people with MS (versus controls) for constipation (OR 6.61; 95% CI 6.00-7.27), visual impairment (OR 4.06; 95% CI 3.33-4.96), chronic pain (OR 3.43; 95% CI 3.18-3.70), migraine (OR 2.38; 95% CI 1.91-2.97), and epilepsy (OR 2.20; 95% CI 1.74-2.77). In contrast, people with MS had a significantly lower prevalence for the majority of cardiovascular (CV) conditions, including atrial fibrillation (OR 0.49; 95% CI 0.36-0.67), chronic kidney disease (OR 0.51; 95% CI 0.40-0.65), heart failure (OR 0.62; 95% CI 0.45-0.85), coronary heart disease (OR 0.64; 95% CI 0.52-0.71), and hypertension (OR 0.65; 95% CI 0.59-0.72).

## Discussion

This study presents findings on the prevalence of comorbidity in patients with MS, based on analysis of a large, geographically and nationally representative Primary Care clinical dataset. Consequently it strengthens the existing evidence already suggesting that comorbidity is common in patients with MS; specifically by reporting on a wider range of comorbidites than any previous comparable study in the field.

Comorbidity of other chronic conditions appears to be common in people with MS [9-11]. Prior research highlights targeted management of such conditions in people with MS as being important [9,10,16]. However, there is an increasing body of evidence suggesting that, around the world, health-care systems, medical education and medical research paradigms are generally not well equipped to address the rising prevalence of people living with multiple conditions. Services have been described as fragmented, and largely orientated towards single-disease models of care, which can be inefficient, unsafe, and can fail to recognise the compound effects of conditions which serves to add to disease burden [13]. Comorbidity in general is associated with reduced functional status, lower quality of life, increased usage of health-care services, and higher mortality rates [13].

People with MS are already known to have reduced health-related quality of life (HRQOL) [17]; and this may be considerably worse in those with comorbidities, with the potential for such conditions to act synergistically to reduce HRQOL [18], which is in keeping with findings in other chronic conditions [10,19]. Comorbidity has previously been demonstrated to delay the time to primary diagnosis in people with MS, possibly by obscuring symptoms that might otherwise be attributed to a new diagnosis [20]. Furthermore, the presence of comorbidity at the time of initial diagnosis has been shown to be associated with increased disability levels [20].

In this study, mental health co-morbidities in people with MS were almost three times as common, compared to the general population, with anxiety and depression being particularly prevalent. This matters because both have been associated with increased suicidal ideation in people with MS, whilst depression is noted to be a key predictor of quality of life, morbidity, and mortality [21]. Anxiety in people with MS, though poorly understood, often increases sharply after initial diagnosis, and is particularly common in female patients [21]. It is known to be associated with greater social dysfunction, increased reporting of physical complaints, excess alcohol usage, and is noted in the literature as being both under-recognised and under-treated [21]. Our findings resonate with the published literature in this sphere, although others have reported even higher levels of depression, but less anxiety [7,22,23]. Speculative links have been drawn between the neuroinflammatory process and the development of depression in people with MS, but the aetiology for depression is complex, likely to be multifactorial, and remains unclear [24], although it is worth noting that depression is a very frequent comorbidity of all physical disease [13].

Recent research has drawn attention to an increased prevalence of bipolar disorder in people with MS [25]. This association was not suggested by the data in our study, which generated a composite (and hence, limited). Prevalance for bipolar disorder (combining schizophrenia, other psychoses, and severe bipolar disorder). However, it is important to note that there is a growing international literature expounding the hypothesis that bipolar spectrum disorders are in fact frequently misdiagnosed as major depressive disorder; most pertinently in the Primary Care setting [26,27]. This is an important point, given that treatment with antidepressants may in fact be harmful in this population, and there are notably high rates of suicide associated with a diagnosis of bipolar disorder.

Recognition and treatment of mental comorbidity in people with MS is important, for the reasons outlined above. Screening in this group, as others have suggested [21,28], may be worthy of consideration. However, the use of such measures in other chronic diseases has been controversial, so any such approach to screening would need careful evaluation [29].

Physical comorbidities were present in nearly two thirds of those with MS, being twice as likely to be found in those without MS, after controlling for age, sex and deprivation. There were notably high levels of nervous system co-morbidities in our study, with chronic pain, migraine and epilepsy all being more than twice as likely to be diagnosed in people with MS, which is in line with the literature. For example, Koch et al. [30] described a prevalence of seizures between 0.5% and 8.3% in people with MS (compared with 1.9% with a diagnosis of active epilepsy in our study (Read Code recorded ever plus anti-epileptic treatment in the previous 12 months)); whilst Kratz et al. [31] described prevalence rates for a self-report survey of chronic pain at up to 50% (as compared to 27.5% of people prescribed 4 or more prescription-only analgesics per year or a pain modifying drug in our study). Chronic pain can impact directly onto levels of psychological stress, with increased utilisation of health care services, diminished quality of life, and more disability [31]. Similarly, a high prevalence of migraine in people with MS has been reported elsewhere [10,32], however, Pakpoor et al. [33] have pointed out in a recent meta-analysis that the relationship between migraine and MS is complicated, with cortical demyelination believed to enhance cortical spreading depression. Visual impairment was over 4 times more likely to be reported amongst people with MS, and this is likely to reflect optic neuritis and its ramifications, which have important implications for both quality of life and lasting visual function. Our high prevalence findings reflect similar results reported from the US [12].

Gastrointestinal diagnoses, such as constipation, dyspepsia, and irritable bowel syndrome (IBS) were more commonly observed in the MS population. Neurogenic bowel dysfunction symptoms are common in people with MS, although whether this represents a separate disease entity is unclear at present [34]. Marrie et al. [9] reported increased prevalence of IBS, in a comparable MS patient population (n = 8983) in the US, at even higher levels than those found in this current sample, but these numbers were based on patient self-report [9].

Of particular note, our study demonstrated a lower prevalence overall for recorded cardiovascular conditions amongst those with MS, when compared to the general population. Cardiovascular pathologies have been reported to lead to greater disability levels in those with MS [35]; and in their own right (cerebrovascular disease, hypertension, hypercholesterolaemia, and diabetes), these conditions are all known to independently be related to both loss of brain mass and function [35]. Research to date has demonstrated conflicting evidence regarding cardiovascular disease amongst people with MS, with a recent systematic review reporting a likely overall increased risk for cardiovascular disease amongst people with MS [36]. The same study also reported that identifying clear cut traditional risk factors (obesity, hypertension, dyslipidaemia, diabetes) that might explain this relationship remains problematic, and suggests that further research in this area is required [36].

Both neurological, and certain gastrointestinal conditions, stand out as over-represented amongst the physical comorbidities. Whether this represents a consequence of MS, its treatments, or of independent comorbidities arising de novo remains unclear. Cardiovascular conditions are remarkably under-represented in this sample, which is particularly notable, given the high rates of cardiovascular disease in Scotland. These findings are comparable to those of Smith et al. [37], who analysed the same dataset and discovered surprisingly low levels of recorded cardiovascular disease amongst patients with Schizophrenia. In both cases, it seems likely that this is at least partly due to under-diagnosis in Primary Care. In the case of people with MS, clinicians and researchers alike should adopt extra vigilance for the potential under-appreciation of cardiovascular dysfunction, given the likely benefits of intervention.

### Strengths and limitations

This study is important given the size and representativeness of the sample in the country with the highest prevalence of MS in the world. It is notable that the prevalence for MS in our sample is comparable (although somewhat higher) to that reported by MacKenzie et al. [38], who reviewed data from the General Practice Research Database from between 1990–2010 (which, although a larger sample in terms of total numbers included, contains a smaller, non-nationally representative cohort of Scottish Primary Care patients – 9.9% of the Scottish population [39]). To our knowledge our data represents the largest clinician recorded dataset for MS comorbidities in the research literature, and reports on the largest number of comorbidities.

Due to the nature of the data available for our analysis, it was not possible to distinguish whether comorbidities were present prior to the diagnosis of MS being applied, or occurred thereafter. The dataset was not designed specifically to study comorbidity in people in MS, and like all routine data analyses it is therefore limited by the quality of the data recording by clinicians, although the use of electronic medical records has been routine in primary care in Scotland since the late 1990s and data recording for common conditions is generally good (however, it is important to note that the positive predictive value for the accuracy of Read Codes in recording diagnoses of MS is far from perfect, with Khan et al. [40] demonstrating rates of around 60% in their systematic review on the topic). Additionally, prescribing data was used to define some conditions in our database, but many drugs have off licence or secondary indications in those with MS (for example to treat spasticity) which may lead to some subtle misclassification. The conditions included in the dataset represent a diverse mixture of morbidities taken from front line clinical practice and prescribing data, and for the purposes of studying MS, it is worth noting that several of these morbidities could potentially be considered as ‘concordant conditions’ (although, to our knowledge, this concept is not well explicated in the MS literature), or even as symptoms of the disease itself. It is also worth pointing out that this descriptive cross-sectional analysis is necesarilly limited by its purposes and the data collected, in that there is no data on disease duration, course, or severity, which precludes both weighting and further analysis by incorporating a specific comorbidity index, beyond that of simply referring to comorbidity count.

## Conclusions

Comorbidity is very common in people with MS, and significantly more common than in the general population, with certain conditions much more likely to be present. Mental health comorbidities, and in particular anxiety and depression, are widespread amongst people with MS. Like the general population, mental health conditions are more likely to be present in people with MS as the number of physical conditions increases, but this relationship is stronger in people with MS. Intriguingly, cardiovascular disease is less likely to be present, although whether this is due to reduced case finding and diagnosis in people with MS or a true difference in prevalence is uncertain. The relationship with cardiovascular disease requires further research, as does the consequences of high rates of mental health comorbidity on physical function and prognosis. Based on experience of physical-mental health comorbidity in conditions like diabetes and coronary heart disease [41,42], interventions are likely to be complex and go beyond drug therapies, and it is likely that interventions tailored to the needs of people with MS will be required. Meantime, clinicians should be alert to the possibility of co-existing mental health conditions and actively treat them if required.

## Competing interests

The authors declare that they have no competing interests.

## Authors’ contributions

SWM, and BG created the dataset on which this study is based. SWM, FM and RS conceived this study. RS led the statistical analysis, with the assistance of GM. All authors contributed to the writing of the report, with RS taking the lead role in this regard. All authors read and approved the final manuscript.

## Pre-publication history

The pre-publication history for this paper can be accessed here:

http://www.biomedcentral.com/1471-2377/14/128/prepub
